# The hidden myocardium: Why intraoperative hypotension should be treated as myocardial injury prevention rather than a transient anaesthetic variable

**DOI:** 10.1177/17504589261454168

**Published:** 2026-06-24

**Authors:** James R Burmeister, Malik Jawad, Tai Metzger, Ismail Zazay, Masab Mansoor

**Affiliations:** 1 Corewell Health William Beaumont University Hospital, Royal Oak, MI, USA; 2 University of Texas Medical Branch, Galveston, TX, USA; 3 Edward Via College of Osteopathic Medicine, Monroe, LA, USA

**Keywords:** Intraoperative hypotension, Myocardial injury after non-cardiac surgery, Perioperative haemodynamics, Mean arterial pressure, Vasopressor therapy, Troponin surveillance, Ischaemic exposure, Autoregulation

## Abstract

Intraoperative hypotension (IOH) has traditionally been regarded as a transient and largely reversible physiological disturbance inherent to the administration of general and regional anaesthesia. However, contemporary perioperative outcomes research consistently suggests that IOH behaves not as a binary, threshold-based phenomenon, but rather as a duration-dependent ischaemic exposure affecting vulnerable end-organs. This manuscript argues that IOH should be reframed as a perioperative type-2 ischaemic syndrome, rather than being treated merely as an expected haemodynamic variable that is easily corrected with fluid boluses or intermittent sympathomimetic administration. Rather than targeting historically derived universal mean arterial pressure (MAP) thresholds alone, perioperative teams should adopt a holistic myocardial-protection framework incorporating MAP, systolic pressure, diastolic pressure, pulse pressure, and markers of organ perfusion. This framework must be grounded in an understanding of the patient’s specific baseline perfusion pressure, an avoidance of cumulative hypotensive burden, and the judicious implementation of titrated vasopressor therapy alongside optimisation of preload, contractility, and tissue perfusion. This reconceptualisation has important implications for intraoperative anaesthetic management, postoperative biomarker surveillance, and the design of interdisciplinary, patient-centred perioperative care pathways.

## Introduction and historical context

Blood pressure is undoubtedly one of the most universally monitored physiological variables in modern perioperative care.

However, arterial pressure is a multidimensional construct rather than a single mean arterial pressure (MAP) value. Systolic blood pressure influences forward flow and stroke work, diastolic blood pressure determines coronary perfusion pressure, and pulse pressure may reflect arterial stiffness and impaired vascular compliance. Contemporary perioperative management therefore requires interpretation of the full arterial waveform rather than reliance on MAP alone (Saugel & Sessler 2021).

From the moment a patient enters the operating theatre to their discharge from the post anaesthesia care unit, haemodynamic status is continuously evaluated. Yet, despite this real-time display and ubiquitous monitoring, intraoperative hypotension is frequently conceptualised as an expected, tolerable, and largely benign consequence of anaesthetic induction and maintenance. Historically, haemodynamic management was guided by foundational cerebral autoregulation studies establishing a general lower limit of safety across broad populations. These early physiological models suggested that as long as systemic pressure remained above a critical nadir, often cited as 50 to 60 mm Hg, end-organ perfusion would be autoregulated and preserved through compensatory local vasodilation.

This concept of a universal autoregulatory threshold was subsequently, and perhaps inappropriately, applied to coronary and renal physiology, even as early cardiovascular researchers noted that myocardial perfusion relies heavily on highly specific diastolic pressure gradients and varies substantially according to individual vascular reserve. The myocardium is exceptionally vulnerable to hypoperfusion because the left ventricle is perfused almost exclusively during diastole. The driving force for this perfusion is the gradient between the aortic diastolic pressure and the left ventricular end-diastolic pressure. When systemic vascular resistance plummets secondary to anaesthetic agents, this critical gradient narrows. For decades, however, these nuanced physiological principles were overshadowed by a reactive clinical approach. A universal target, commonly an MAP of 65 mm Hg, was historically deemed sufficient for many patients. However, subsequent evidence suggests that identical MAP values may conceal markedly different systolic, diastolic, and flow states between individuals.

## The shift to recognising end-organ injury

The entrenched assumption that transient intraoperative hypotension (IOH) is a benign, easily reversible event began to erode substantially as large, highly powered retrospective analyses successfully linked brief intraoperative hypotensive episodes to severe, life-altering adverse outcomes, initially highlighting the poorly understood risk of perioperative stroke (Bijker et al 2012). These early studies demonstrated that the brain, despite its robust autoregulatory mechanisms, was not immune to the profound haemodynamic shifts occurring during surgery.

However, the landscape of perioperative medicine underwent an important paradigm shift with the identification and classification of myocardial injury after non-cardiac surgery (MINS). Researchers established MINS as a highly prevalent and prognostically significant event, proving it to be independently and strongly associated with 30-day mortality (Devereaux et al 2012). Unlike classic type-1 myocardial infarctions, which are driven by acute plaque rupture and thrombosis, MINS is predominantly a type-2 ischaemic event driven by a severe mismatch between myocardial oxygen supply and demand. In the operating theatre, this mismatch is most frequently precipitated by hypotension, often exacerbated by concurrent tachycardia or surgical anaemia.

Subsequent large-scale observational studies demonstrated that even highly brief periods, specifically durations of just 1 to 5 min, of MAP reduction below absolute thresholds were significantly associated with a measurable increase in the risk of myocardial injury (Walsh et al 2013). It quickly became apparent to the scientific community that this hypotensive damage was not isolated strictly to the heart. Similar brief ischaemic exposures were associated with the development of postoperative acute kidney injury, suggesting a systemic vulnerability to transient hypoperfusion (Sun et al 2015). Because perioperative myocardial injury frequently presents without classic ischaemic symptoms such as chest pain or dyspnoea, partly due to the residual effects of analgesics and sedatives, routine postoperative troponin screening was increasingly advocated as a mandatory surveillance tool to detect these otherwise clinically silent yet significant events (Abbott et al 2018).

Emerging data also suggest that isolated maintenance of MAP may be insufficient when accompanied by low systolic pressure, widened pulse pressure, or reduced stroke volume. In such states, perfusion pressure may appear preserved while effective organ blood flow remains compromised. This distinction is particularly relevant in older adults, patients with arterial stiffness, and those with heart failure.

## Hypotensive burden: depth, duration, and individualisation

As data matured and computational analyses of electronic health records became more sophisticated, research firmly established a continuous, exposure-dependent relationship between the severity of hypotension and postoperative mortality, strongly suggesting that haemodynamic risk is not a binary phenomenon (Salmasi et al. 2017). A patient does not abruptly cross from absolute safety to absolute danger the moment their MAP drops from 66 to 64 mm Hg. Rather, the risk accumulates in a dose-dependent manner based on the severity and duration of the hypoperfusion.

This continuous risk profile prompted investigators to challenge the ‘one-size-fits-all’ approach and launch investigations into individualised haemodynamic targets. Landmark clinical trials demonstrated that maintaining a patient’s MAP within a tight variance, specifically within 10% of their unique preinduction baseline, significantly reduced the incidence of postoperative organ dysfunction when compared to standard universal targets (Futier et al 2017). A patient with chronic, poorly controlled hypertension and a resting MAP of 105 mm Hg has likely undergone significant vascular remodelling. For this individual, an MAP of 65 mm Hg represents a substantial reduction in perfusion pressure, well below their right-shifted autoregulatory curve (Yin et al 2025).

Consequently, the concept of ‘hypotensive burden’ emerged as a central theme in the anaesthesia literature. Researchers demonstrated that time-weighted average MAP, which accounts for the total area under the curve of hypotensive exposure, provided far superior risk prediction than intermittent, single-threshold measurements (Sessler et al 2018). Furthermore, the methodology of measurement was called into question. Studies utilising continuous invasive blood pressure monitoring via arterial cannulation revealed that intermittent non-invasive blood pressure cuffs, typically cycling every 3 to 5 min, frequently missed deep, transient hypotensive valleys (Maheshwari et al 2018). Comprehensive meta-analyses subsequently confirmed that the cumulative area under a critical perfusion curve, integrating both the absolute depth and the total duration of the hypotensive event, is the primary driver of postoperative cardiovascular morbidity (Wesselink et al 2018).

## Beyond MAP: flow, pulse pressure, and coronary perfusion

Although MAP remains a practical bedside target, it should not be interpreted in isolation (Saugel et al 2024). Coronary perfusion occurs predominantly during diastole, making diastolic hypotension especially relevant to myocardial oxygen supply. Conversely, inadequate systolic pressure may reduce stroke volume and systemic flow, particularly in patients with impaired ventricular function. Abnormal pulse pressure may indicate reduced vascular compliance or low stroke volume, both of which have been associated with adverse perioperative outcomes. A modern haemodynamic strategy should therefore integrate pressure targets with assessment of cardiac output, volume responsiveness, and tissue perfusion markers such as lactate, urine output, capillary refill, and echocardiographic findings (Scott 2024).

## The vulnerable patient and the causality debate

Understanding the true impact of IOH requires an appreciation of the changing demographic of the modern surgical patient. The contemporary surgical population is increasingly characterised by advanced age, systemic endothelial dysfunction, and a high burden of occult cardiovascular disease (Benjamin et al 2019). Patients presenting for major non-cardiac surgery frequently suffer from left ventricular hypertrophy, undiagnosed coronary artery disease, and diabetic microvascular complications. In these vulnerable populations, the profound systemic vasodilation induced by propofol and volatile anaesthetic agents, combined with a severely impaired autoregulatory reserve, creates conditions that strongly predispose to myocardial supply-demand mismatch. This risk may be amplified when vasopressor therapy restores numeric arterial pressure without adequately improving microvascular flow or cardiac output. Thus, what appears on a standard vital signs monitor as a short-lived decrease in blood pressure often constitutes clinically meaningful ischaemic time for the myocardium at the cellular level.

It must be explicitly acknowledged, however, that the largely observational nature of much of the IOH research has sparked a legitimate and ongoing academic debate. Sceptics and cautious interpreters of the data rightfully question whether hypotension is a direct mechanistic cause of tissue injury, or merely a secondary proxy for underlying illness severity, surgical bleeding, and general physiological frailty (Liem et al 2020). The counterargument posits that sicker patients are simply more prone to vasoplegia, and that the hypotension is a symptom of their impending morbidity rather than the cause. However, while definitive, isolated causality remains highly complex to prove in a multifaceted surgical environment, the clinical consensus overwhelmingly favours a precautionary, proactive approach. Even if hypotension is partially a marker of frailty, allowing a frail patient’s perfusion pressure to collapse is physiologically unsound and clinically dangerous.

## Moving towards a myocardial protection model

The theoretical debates regarding causality are increasingly being settled by actionable clinical evidence. Recent interventional trials have strongly reinforced the value of strict, individualised blood pressure control to mitigate perioperative risk, showing that active, protocolised avoidance of hypotensive burden may improve patient outcomes (Saugel et al 2025). Reframing IOH as preventable ischaemic time necessitates a fundamental and cultural shift in perioperative care. Anaesthetic practitioners must transcend the traditional role of simply facilitating surgical conditions and must serve as the primary guardians of myocardial and end-organ perfusion.

Implementing this myocardial protection model requires several concrete changes in daily practice. First, preoperative assessments must establish accurate, resting baseline blood pressures, rather than relying on anxiety-driven measurements taken in the immediate preoperative holding area. Second, intraoperative targets must be dynamically referenced against these baselines. Third, practitioners should move beyond reactive rescue boluses towards proactive haemodynamic management that may include carefully titrated vasopressor infusions, rational fluid therapy, and treatment of reversible causes such as anaesthetic depth, vasodilation, bleeding, or ventricular dysfunction. Finally, health care systems must standardise the integration of highly granular intraoperative haemodynamic data with routine postoperative cardiac surveillance, including scheduled high-sensitivity troponin assays for at-risk cohorts.

## Conclusion

Vasopressors must be used thoughtfully. Excessive vasoconstriction may increase ventricular afterload, worsen myocardial work, precipitate heart failure in susceptible patients, or impair regional microcirculatory perfusion despite restoration of macrocirculatory pressure. The optimal strategy is therefore not maximal pressure generation, but balanced restoration of both pressure and flow. Emerging predictive technologies may further reduce hypotensive burden through earlier identification of haemodynamic deterioration (Mukkamala et al 2025).

By adopting an individualised protection framework that balances pressure targets with effective organ blood flow, interdisciplinary teams can illuminate the ‘hidden myocardium’ and reduce the silent burden of perioperative cardiac injury.

## Data Availability

Not applicable.
